# Involvement of a Quorum Sensing Signal Molecule in the Extracellular Amylase Activity of the Thermophilic *Anoxybacillus amylolyticus*

**DOI:** 10.3390/microorganisms9040819

**Published:** 2021-04-13

**Authors:** Annabella Tramice, Adele Cutignano, Annalaura Iodice, Annarita Poli, Ilaria Finore, Giuseppina Tommonaro

**Affiliations:** National Research Council of Italy, Institute of Biomolecular Chemistry, 34- 80078 Pozzuoli, Italy; atramice@icb.cnr.it (A.T.); acutignano@icb.cnr.it (A.C.); annalaura94@live.com (A.I.); apoli@icb.cnr.it (A.P.); ilaria.finore@icb.cnr.it (I.F.)

**Keywords:** *Anoxybacillus amylolyticus*, extremophiles, quorum sensing, α-amylase, trans-cinnamaldehyde, UPLC/MRM

## Abstract

*Anoxybacillus amylolyticus* is a moderate thermophilic microorganism producing an exopolysaccharide and an extracellular α-amylase able to hydrolyze starch. The synthesis of several biomolecules is often regulated by a quorum sensing (QS) mechanism, a chemical cell-to-cell communication based on the production and diffusion of small molecules named “autoinducers”, most of which belonging to the *N*-acyl homoserine lactones’ (AHLs) family. There are few reports about this mechanism in extremophiles, in particular thermophiles. Here, we report the identification of a signal molecule, the *N*-butanoyl-homoserine lactone (C4-HSL), from the milieu of *A. amylolyticus*. Moreover, investigations performed by supplementing a known QS inhibitor, *trans*-cinnamaldehyde, or exogenous C4-HSL in the growth medium of *A. amylolyticus* suggested the involvement of QS signaling in the modulation of extracellular α-amylase activity. The data showed that the presence of the QS inhibitor *trans*-cinnamaldehyde in the medium decreased amylolytic activity, which, conversely, was increased by the effect of exogenous C4-HSL. Overall, these results represent the first evidence of the production of AHLs in thermophilic microorganisms, which could be responsible for a communication system regulating thermostable α-amylase activity.

## 1. Introduction

*Anoxybacillus amylolyticus* (strain MR3C^T^=ATCC BAA-872^T^) is a moderate thermophilic Gram-positive rod and facultative anaerobe. Exhibiting optimum growth temperature at 61 °C, it is able to grow between 45 and 65 °C and at pH 5.6. It is able to produce an exopolysaccharide and to synthesize an extracellular constitutive amylolytic activity which digests starch. The maximum amylolytic activity is expressed in the stationary growth phase [1]. Amylases belong to families of enzymes used in several biotechnological and industrial processes (e.g., starch processing, sugar production) [2,3], and in textile and pharmaceutical industries [4]. They can be obtained from different sources, including plants, animals, and microorganisms, although microbial enzymes are generally preferred, due to their advantages such as efficient production, stability, and cost-effectiveness [5,6]. The ability of *A. amylolyticus* to produce extracellular amylase enzymes is related to its role in hydrolyzing complex organic matter and generating nutrients. Purified extracellular α-amylase from *A. amylolyticus* showed a molecular weight of about 60 KDa, as confirmed by gel filtration chromatography an SDS-PAGE; the latter additionally suggested a single polypeptide chain structure [7]. Several bacterial species were disclosed to have a quorum sensing mechanism, acting at gene expression level, to regulate a wide variety of physiological activities, e.g., motility, biofilm development, growth inhibition, virulence expression, and plasmid conjugation [8]. At the basis of QS there is synthesis by these microorganisms and the subsequent release in their extracellular milieu of small diffusible compounds, named auto-inducers (AIs). The best known QS mechanism relies on the synthesis of *N*-acyl-homoserine lactones (AHLs) (AI-1 QS), but other QS systems based on different signal molecules (4,5-Dihydroxy-2,3-pentanedione (DPD), autoinducer-1 (CAI-1), diketopiperazines (DKPs)) were found among various microorganisms [9,10]. Despite numerous reports on well-characterized cell-to-cell communication in a wide variety of microorganisms, QS is still poorly explored in extremophiles, thus requiring further investigation. In fact, in extremophiles QS could also play a key role in the production of extracellular metabolites (such as enzymes, exopolysaccharides, and lipids) that are attractive for many biotechnological applications in different fields (pharmacology, ecology, molecular biology, and medicine) [11,12,13].

In this paper, we report the detection of a QS system based on *N*-butanoyl-homoserine lactone (C4-HSL) in the extremophilic microorganism *A. amylolyticus*. Moreover, the results on the use of *trans*-cinnamaldehyde (CIN), a known inhibitor of QS [14], and of C4-HSL supplementation during the microbial growth on the extracellular amylolytic activity of *A. amylolyticus* are discussed.

## 2. Materials and Methods

### 2.1. Chemicals

*N*-butanoyl-(C4-HSL), *N*-hexanoyl-(C6-HSL), *N*-decanoyl-(C-10-HSL), *N*-dodecanoyl- (C12-HSL), *N*-3-oxobutyryl-(3-oxo-C4-HSL), *N*-3-oxohexanoyl-(3-oxo-C6-HSL), *N*-3-oxodecanoyl-(3-oxo-C10-HSL), *N*-3-oxotridecanoyl-(3-oxo-C13-HSL), *N*-3-hydroxyhexanoyl-(3-OH-C6-HSL), *N*-3-hydroxyoctanoyl-(3-OH-C8-HSL), *N*-3-hydroxydecanoyl-(3-OH-C10-HSL) homoserine lactone, and trans-cinnamaldehyde were purchased from Sigma-Aldrich, Milano, Italy. LC-MS grade acetonitrile (ACN) was purchased from Merck (Darmstadt, Germany). Water for LC-MS analysis was obtained by a milliQ apparatus (Millipore, Milano, Italy). Standard stock solutions for quantitative analyses were prepared by dissolving C4-HSL in methanol (MeOH) at 100 µg mL^−1^, stored at –20 °C. Calibration solutions were prepared by diluting the stock solution to the desired concentration (0.2, 1, 5, 20, and 200 ng mL^−1^) with MeOH. Merck company (Darmstadt, Germany) furnished silica gel plates for thin layer chromatography (TLC). Compounds on TLC plates were visualized by charring with α-naphthol reagent. The TLC solvent system EtOAc/AcOH/2-propanol/HCOOH/H_2_O (25:10:5:1:15 *v*/*v*) was used for the visualization of oligosaccharide mixtures of up to six units. Protein concentration was determined by the method of Bradford [15], using bovine serum albumin as standard. Chemical determination of reducing sugar amount was made by the Bernfeld method (3,5 dinitrosalicylic acid, DNS assay) [16], using a glucose-based calibration curve.

### 2.2. A. amylolyticus Cultivation

*A. amylolyticus* (strain MR3C^T^ =ATCC BAA-872^T^) was grown in Y_N_ DSM medium containing the following components: yeast extract 0.6% *w*/*v*, NaCl 0.6% *w*/*v*, and Agar 1.8% *w*/*v* (for plate preparation) at 60 °C. The pH of the medium was adjusted at 5.6 by using 0.2 M HCl [1]. C4-HSL standard was added to the medium at a concentration of 10 µM, and CIN was added to the medium to give final concentrations of 1 and 3 mg mL^−1^ (7.5 and 22 mM, respectively). Growths were carried out for 24 h and monitored both by evaluating their optical density (O.D., A_540nm_) and by observation at phase-contrast microscopy (Zeiss). Moreover, cell viability was checked by means of a serial dilution plating method.

### 2.3. Extraction of Spent Medium of A. amylolyticus

Spent medium (100 mL) from stationary phase cultures of *A. amylolyticus* was centrifuged at 13,848 *g* for 40 min. Pellets were stored at −20°C for further investigation, while supernatants were extracted with ethyl acetate (1:1 *v*/*v*; twice) and next dried in vacuum at T < 40°C. The obtained extract was dissolved with methanol to a final concentration of 0.6 mg mL^−1^ and directly tested for the detection of QS signal molecules by means of TLC-overlay assay or diluted 1:10 for LC-MS/MS analysis.

### 2.4. Identification of Autoinducer 

#### 2.4.1. TLC-Overlay Assay

Supernatant extract and standards (3-oxo-C6-HSL 10 μM and 3-oxo-C10-HSL 400 μM) were applied to RP-C18 thin-layer chromatography (TLC) plates (20 × 20 cm; VWR International), and developed by using as mobile phase 60% (*v*/*v*) aqueous methanol. The TLC plates were overlaid with 100 mL of AGTN [17] soft agar (0.5% *w*/*v*) supplemented with 0.5% glucose, 40 μg mL^−1^ X-Gal (5-bromo-4-chloro-3-indolyl-beta-D-galactopyranoside), antibiotics (streptomycin, 50 μg mL^−1^ and tetracycline, 4 μg mL^−1^), and the biosensor *Agrobacterium tumefaciens* NTL4 (pCF218; pCF372) able to detect AHLs with medium chain lengths [18]. The TLC plates were kept in a sterile container and incubated at 30 °C for 24–48 h.

#### 2.4.2. LC-MS/MS Analysis

LC-MS analyses were performed according to Abbamondi et al., 2016 [11], with slight modifications. Briefly, chromatographic runs were acquired on an Acquity UPLC System (Waters, Milford, MA, USA) coupled to a 3200 API Triple Quadrupole mass spectrometer (Sciex, Foster City, CA, USA) with aTurbo VTM interface equipped with a turbo ion spray probe used in positive ion mode and on a Acquity UPLC BEH C_18_ column (100 × 2.1 mm, i.d. 1.7 µm, Waters, Milford, MA, USA). A water/ACN (9:1, *v*/*v*) mixture was used as eluent A, and ACN (100%) as eluent B. A linear gradient profile was programmed from 100% A to 100% B in 1.0 min and remained constant over 3.0 min, followed by a re-equilibration step of 5 min. Separations were performed at a temperature of 60°C, using a flow rate of 0.7 mL min^−1^ and an injection volume of 2 µl.

A multiple reaction monitoring (MRM) experiment was used to collect data, by setting the following source parameters: curtain gas (N_2_): 20 psi; ion source gas (GS1): 55 psi; turbogas (GS2): 70 psi; desolvation temperature: 550 °C; collision activated dissociation gas (CAD): 4 a.u.; and ion spray voltage: 5500 V. The ions monitored in Q1 included the parent AHL [M+H]^+^ ion, while in Q3 the lactone moiety at *m*/*z* 102 was monitored. Analyst software (version 1.6.2; SCIEX) was used for the data acquisition and analysis.

### 2.5. A. amylolyticus α-amylase Assay

Extracellular α-amylase activity was recovered from the stationary 24 h bacterial growths by centrifugation at 8309 *g* for 30 min and at room temperature. The cell-free supernatant was concentrated by ultrafiltration (Amicon system) on a Millipore Ultrafiltration membrane with a 30 kDa cut-off. Enzymatic activity was recovered in the retentate solution; it was tested for its protein content [15] and α-amylase presence [16]. The α-amylase activity was assayed referring to and modifying as previously described by Finore et al., 2014 [19]. All values given are averages of three assays.

A solution of 2.5 mg mL^−1^ starch in 100 mM sodium acetate buffer with a pH 5.6 was put at 60 °C under magnetic stirring and incubated with a fixed protein amount of several extracellular enzymatic solutions from different bacteria growth performed with CIN, C4-HSL, and a bacterial growth selected as a reference (blank experiment), as described in Section 2.2.

For each enzymatic digestion, a fixed starch/ total protein weight ratio (substrate/enzyme, s/e) of 25.64 was adopted, which corresponded to 0.79 mg of the total protein for 20 mg of starch substrate. Enzymatic reactions were monitored over the time: aliquots of 0.4 mL of reaction were withdrawn at 0 min, 5 min, 10 min, 15 min, 20 min, 30 min, 45 min, 1h, 2h, 3h, 4h, 5h, 6h, 7h, and 24 h; the processes were stopped in an ice-bath for 5 min and the samples were stored at −20 °C overnight. Next, 0.100 mL of each sample was assayed for the glucose equivalent production (DNS assay). These tests were conducted in triplicate and the calibration curve of glucose was elaborated in a concentration interval of 0.05:1 mg mL^−1^. Reactions were also monitored by TLC.

One unit of amylase activity was defined as the nanomoles of glucose equivalent which were produced in a minute by 1 mg of the enzymatic solution.

Results were expressed as mean values ± standard error (ES). Means, ES, calibration curves, and linear regression analyses (R^2^) were obtained using Microsoft Excel 2016 (Microsoft Corporation, Redmond, WA, USA).

## 3. Results

The growth of *A. amylolyticus* cultivated in different conditions was monitored by O.D. (A_540nm_) measurement after 24 h and the following values were registered: 0.586 ± 0.015, blank; 0.558 ± 0.021, 1 CIN; 0.560 ± 0.012, 3 CIN; and 0.540 ± 0.019, C4-HSL (10 µM). The O.D. values were supported by the observation at phase-contrast microscopy that showed, after 24 h, similar growth condition (motile and living bacteria) in the blank, 1 CIN, 3 CIN, and C4-HSL. Furthermore, the serial dilution plating method showed non-significative differences between the control and the cultures in presence of CIN and C4-HSL The presence of the QS inhibitor CIN and of C4-HSL standard did not affect the growth of *A. amylolyticus*.

### 3.1. Identification of the Autoinducer

The ethyl acetate extract (100 µL of a solution 0.6 mg mL^−1^ in methanol) of spent medium of *A. amylolyticus* grown in standard conditions (blank) was analyzed by means of TLC overlay assay, with the biosensor *Agrobacterium tumefaciens* NTL4 (pCF218; pCF372) able to detect AHLs with medium chain lengths [18] with the aim to assess the presence of signal molecules. A blue spot with an R_f_ of 0.4 was detected on the TLC, suggesting the occurrence of AHLs with short acyl chains.

**Figure 1:**
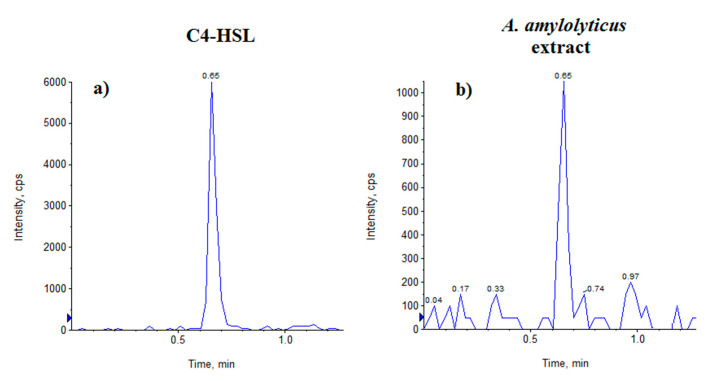
Representative LC-MRM (*m*/*z* 172 > 102) chromatograms of **a**) *N*-butanoyl-homoserine lactone (C4-HSL) standard and **b**) *Anoxybacillus amylolyticus* extract.

The identification of AHL at the molecular level was performed in the bacterial extract by UPLC-MS/MS analysis monitoring diagnostic MRM transitions associated to HSL standards according to the MS method previously reported, here slightly modified [10]. MS data disclosed the occurrence in the bacterial extract of C4-HSL, as confirmed by both specific MRM transition 172 > 102 and Rt (0.65min) compared with the standard (Figure 1). A calibration curve built with five calibration points (y = 3373.1 x –328.30; R^2^ = 0.9997) was used to assess the absolute amount of the signal metabolite (12 ng) in the whole bacterial culture (100 mL).

**Figure 2:**
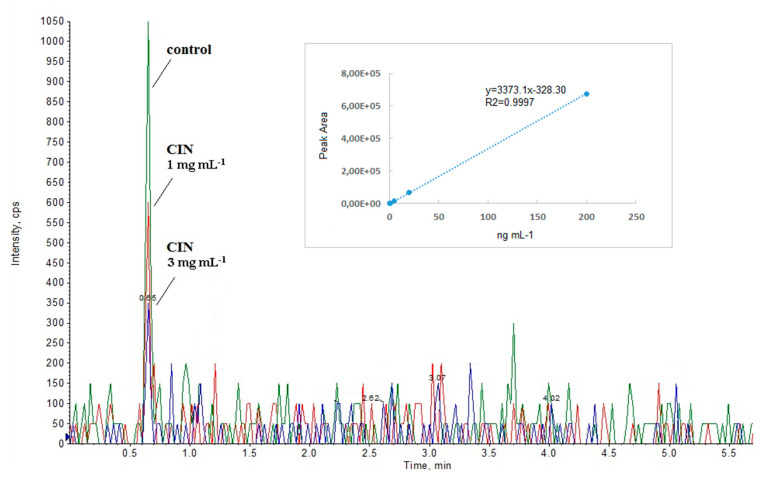
Representative LC- MRM chromatograms for *m*/*z* 172 > 102 transition attributed to C4-HSL in the control culture grown in standard conditions (control) and with 1 and 3 mg mL^−1^ *trans*-cinnamaldehyde (CIN). The inset shows the calibration curve built by plotting peak area vs standard C4-HSL concentration.

As regards the extracts of culture with 1 or 3 mg mL^−1^ of CIN subsequently produced, the addition of this molecule to the culture media induced an appreciable reduction in bacterial culture (100 mL) of C4-HSL levels measured in the above LC-MS conditions as 6.5 ng and 3.4 ng, respectively (Figure 2).

### 3.2. A. amylolyticus α-amylase Monitoring in Different Bacterial Growth Conditions

Extracellular α-amylase activity was investigated with the aim to evaluate the correlation between the expression of this enzyme and the presence in the growth media of inhibitor molecules or signal molecules of bacterial quorum sensing (QS), such as CIN and C4-HSL, respectively. These compounds were added at different concentrations into the bacterial culture mixtures, as reported in materials and methods. Enzymatic solutions obtained after centrifugation and ultrafiltration were assayed for their total protein content. An evaluation of the total protein amount suggested a possible influence of CIN in the bacterial metabolism with a reduction of the recovered extracellular proteins, although the final concentrations added to the medium were of 7.5 and 22 mM and in general at the limit of those previously reported for other bacteria [19]. In both experiments the concentration of the recovered extracellular protein substantially decreased.

These results would suggest an influence of CIN in bacterial metabolism, but there was no correlation between CIN toxicity and bacterial growth.

All the starch degradation experiments were performed by using the same amount of the extracellular total protein.

**Figure 3:**
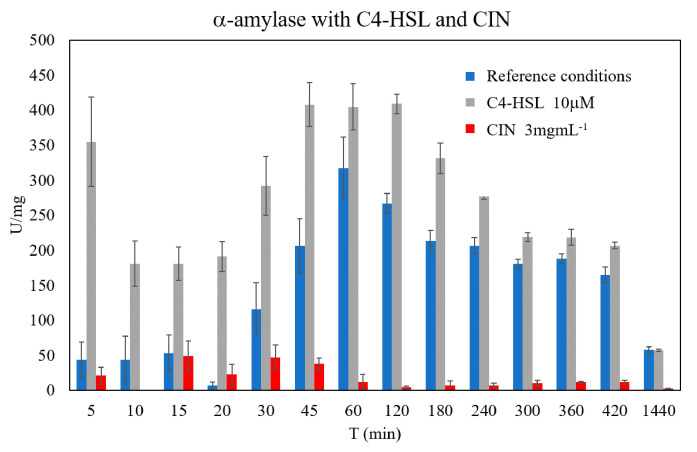
α-amylase activity from a culture containing 10 µM C4-HSL and CIN 3 mg mL^−1^, compared with the activity expressed in a bacterial culture performed under standard medium conditions (reference conditions).

In Figure 3, starch digestions with α-amylase activity in the enzymatic solutions recovered from bacterial cultures, carried out in presence of C4-HSL and CIN, were compared with the activity expressed in a bacterial culture performed under standard conditions (blank culture). Exogenous C4-HSL and CIN were added to the growth medium at 10 µM and 3 mg mL^−1^, respectively.

This activity was measured in terms of the reduction of the glucose equivalent produced over time (Section 2.5).

In addition, the thin-layer chromatography (TLC) technique was used for monitoring the hydrolytic products of the α-amylase enzyme on the starch (Figure S1 of the Supplementary Materials) and the percentage of starch hydrolyzed was calculated (Figure S2 of the Supplementary Materials).

For the experiment with C4-HSL, the enzymatic digestion pattern was similar to that of the experiment under standard conditions; at 45–60 min the maximum enzymatic hydrolytic potential for the production of maltooligosaccharides was recorded, in agreement with the results previously reported by Finore et al. in 2014 [7]. It is important to emphasize that when C4-HSL was present the bacterial medium the α-amylase activity appeared to increase (Figure 3). It was evident that at the beginning of the starch digestion (from 5 to 15 min) specifically this activity increased by 8 to 4 times: 180.08 U mg^−1^ at 10 min in presence of C4-HSL, and 43.62 U mg^−1^ in the blank conditions at the same time mark. However, this increase was significant up to 4 h of reaction, when the percentage of hydrolyzed starch was at 47% for the process with the exogenous C4-HSL and 35% for the reaction with the α-amylase recovered from the standard bacterial growth (Figure S2).

At 24 h, a prevalent accumulation of smaller and less hydrolysable oligosaccharides (end products maltohexaose, maltopentaose, maltotriose, and maltose) (Figure S1), and a physiological enzymatic denaturation could justify a decrease of activity at 57–58 U mg^−1^ in both enzymatic processes; at this final reaction time, 57% of the starch was depolymerized in both cases (Figure S2).

Differently, when CIN was added into the bacterial medium, the α-amylase activity decreased. The best results were recorded when CIN was used at the highest concentration of 3 mg mL^−1^, as shown in Figure 3. In this case, the enzymatic activity was remarkably reduced, especially starting from 45–60 min: resulting in 37.86 and 12.17 U mg^−1^ at 45 min and 60 min, respectively, and a minor production of smaller oligosaccharides was observed with only ~1% of the degraded starch (see also Figures S1 and S2). A partial decrease of enzymatic activity was observed also for the experiment with CIN at 1 mg mL^−1^. Indeed, in this case, in the first 60 min the trend of enzymatic activity was unusual, and only after 60 min was the effect was appreciably evident. In fact, at 1 h, a value of 197.05 U mg^−1^ was measured, which resulted in an appreciably lower value than that obtained under standard conditions (317.47 U mg^−1^) and higher than the value with CIN at 3 mg mL^−1^ (12.17 U mg^−1^) (Figure S3). These data would suggest a dependence of the α-amylase activity on the used CIN concentration in the media. It should be noted that this is the first study regarding the possible involvement of CIN on a thermophilic α-amylase activity: how enzymatic activity could be modulated by this compound will be investigated in the future, after the purification of the enzymatic protein.

## 4. Discussion

In the search for new biomolecules useful for biotechnological applications, special attention has been paid to extremophilic microorganisms, due to their ability to produce unusual biomolecules (such as thermostable enzymes and exopolysaccharides) as one of their adaptive mechanisms to harsh environmental conditions. Molecules produced under extreme conditions possess features that have proved useful in numerous industrial applications, with the effect of increasing the efficiency and/or the sustainability of production processes [13]. The biosynthesis of many of these biomolecules is regulated by quorum sensing mechanisms through the production, diffusion, and detection of small molecules named autoinducers, mainly represented by AHLs. Since this molecular communication system in extremophiles is still poorly studied, researchers have increasingly focused on this topic. The isolation and identification of AHLs in extremophiles have been reported in halophiles, in particular the genus *Halomonas*. Some AHLs from *H. anticariens* FP35(T) were identified: *N*-butanoyl-homoserine lactone (C4-HSL), *N*-hexanoyl-homoserine lactone (C6-HSL), *N*-octanoyl-homoserine lactone (C8-HSL), and *N*-dodecanoyl-homoserine lactone (C12-HSL) [20], and their QS systems were composed of the *luxR/luxI* homologues: *hanR* (the transcriptional regulator gene) and *hanI* (the AHL synthase gene) [21]. An AHL with an unsubstituted acyl side chain length of C16 was identified in *Halomonas smyrnensis* AAD6, a moderately halophilic, exopolysaccharide-producing bacterium isolated from a saltern area in the Aegean region of Turkey The authors hypothesized that the growth-phase dependent production of the EPS could be regulated by QS [11,22]. In cultures of the acidophile *Acidithiobacillus ferrooxidans,* the detection of an AHL with an unsubstituted acyl side chain length of C14 has been reported [23]. In Archaea the presence of a quorum sensing mechanism, based on AHLs and other type of signal molecules, has also been described [10,24,25,26]. There are few records in the literature on the presence of QS molecules in thermophiles and most of them reported indirect evidences [27,28,29,30]. In this paper, we report for the first time the identification of a signal molecule, belonging to the AHLs group, identified as *N*-butanoyl-homoserine lactone (C4-HSL), from cultures of the thermophilic bacterium *A. amylolyticus*. We also assessed the absolute amount of the signal metabolite (12 ng) in the whole bacterial culture (100 mL), and found it to be very low compared with the common concentrations found in pure cultures of non-extremophiles microorganisms [31]. Since *A. amylolyticus* is able to produce an extracellular amylase enzyme, we investigated the involvement of the QS mechanism in the extracellular amylolytic activity, comparing the C4-HSL level in a control growth, in presence of a QS inhibitor, *trans*-cinnamaldehyde (CIN) [14], or of exogenous C4-HSL.

In effect, the presence of the QS inhibitor CIN in the medium decreased the synthesis of the autoinducer C4-HSL, as revealed by LC-MS analysis, and suggested its possible involvement in amylolytic activity; conversely, the presence of extra C4-HSL resulted in an appreciable increase of extracellular amylase activity. However, QS may affect other protein(s) and its impact on α-amylase activity could be indirect, resulting from the assay being based on a fixed amount of extracellular protein.

The regulatory link between QS signals and extracellular biomolecules (enzymes, exopolysaccharides, and virulence factors) production is a possible target to mitigate the virulence of pathogens, such as *Pseudomonas aeruginosa*, and for enhancing the production of attractive and valuable molecules [32,33]. Our results showed that the production of α-amylase by *A. amylolyticus* could be regulated by a quorum sensing mechanism based on AHLs, and that it could be increased by the supplementation of C4-HSL in the growth medium. In fact, under the latter growth conditions, we noted an increase in α-amylase activity of up to 50% in the first 45 min of the starch digestion processes, with respect to the standard growth conditions. This result is particularly significant because α-amylase produced by the thermophilic *A. amylolyticus* represents a thermostable enzyme able to digest cellulose, hemicellulose, and starch at high temperatures, working conditions that are often present in industrial processes.

## 5. Conclusions

In conclusion, our results provide direct evidence of AHL production in thermophilic microorganisms, which could be responsible for a communication system. In particular, by using highly sensitive mass spectrometry-based approaches, we identified in *A. amylolyticus* C4-HSL as autoinducer [11,34]. Moreover, biochemical studies highlighted the correlation of C4-HSL levels with the extracellular amylolytic activity of *A. amylolyticus*. Overall, our data suggest the occurrence of a metabolic network including a QS mechanism and glycosidase activities, and the regulation of QS as a possible strategy to implement the sustainable production of valuable biomolecules in different industrial sectors.

## Data Availability

The data presented in this study are available on request from the corresponding author.

## Supplementary Materials

The following are available online at https://www.mdpi.com/article/10.3390/microorganisms9040819/s1, Figure S1: TLC analysis of starch digestion at 1 and 24 h, Figure S2: % of starch hydrolysis by using α-amylases from bacterial growth in standard condition, with CIN (3 mgmL^−1^) and C4-HSL (10 μM), Fugure S3: α-Amylase activity from a culture containing 1 mg mL^−1^ of CIN.
