# Supplementary figures and images for: Involvement of a Quorum Sensing Signal Molecule in the Extracellular Amylase Activity of the Thermophilic Anoxybacillus amylolyticus
Source: Microorganisms. 2021 Apr 13;9(4):819. doi: 10.3390/microorganisms9040819 (PMC8068869; doi:10.3390/microorganisms9040819)

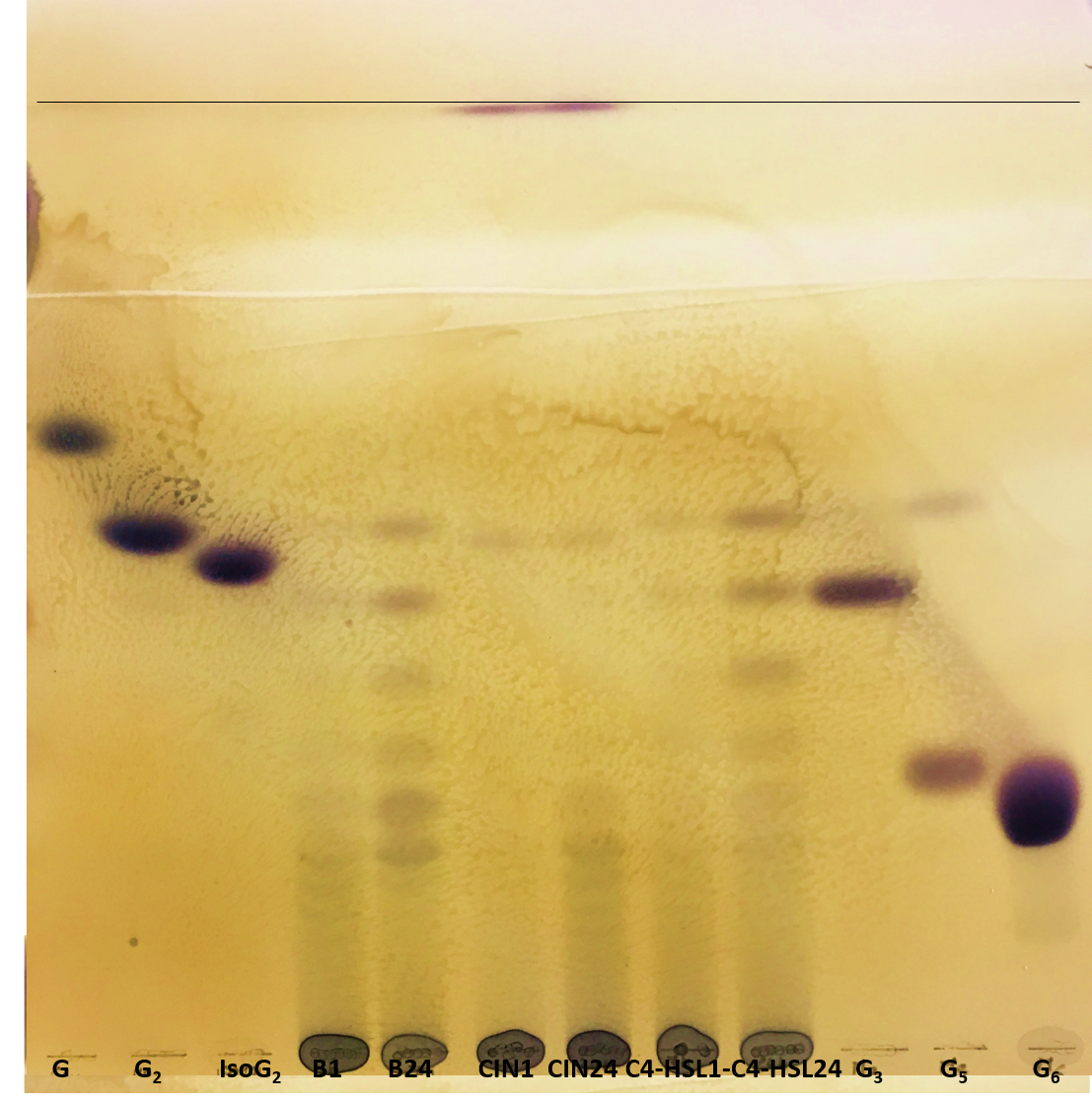

Supplement: Supplementary file 1 [file microorganisms-09-00819-s001.zip › Figure S1.png]

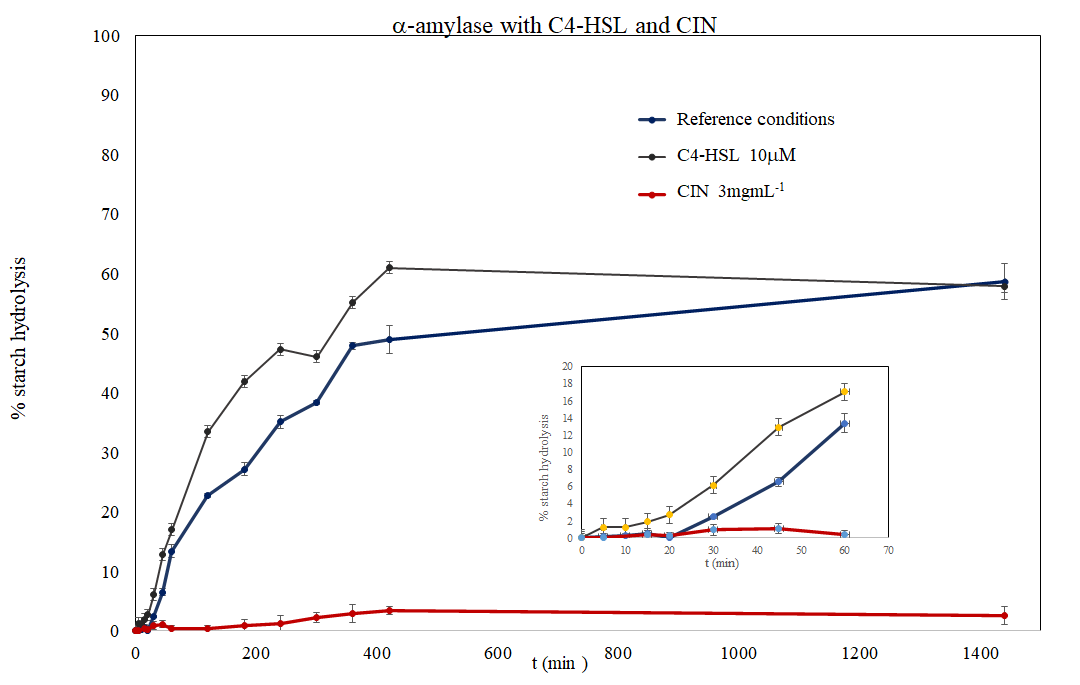

Supplement: Supplementary file 1 [file microorganisms-09-00819-s001.zip › Figure S2.png]

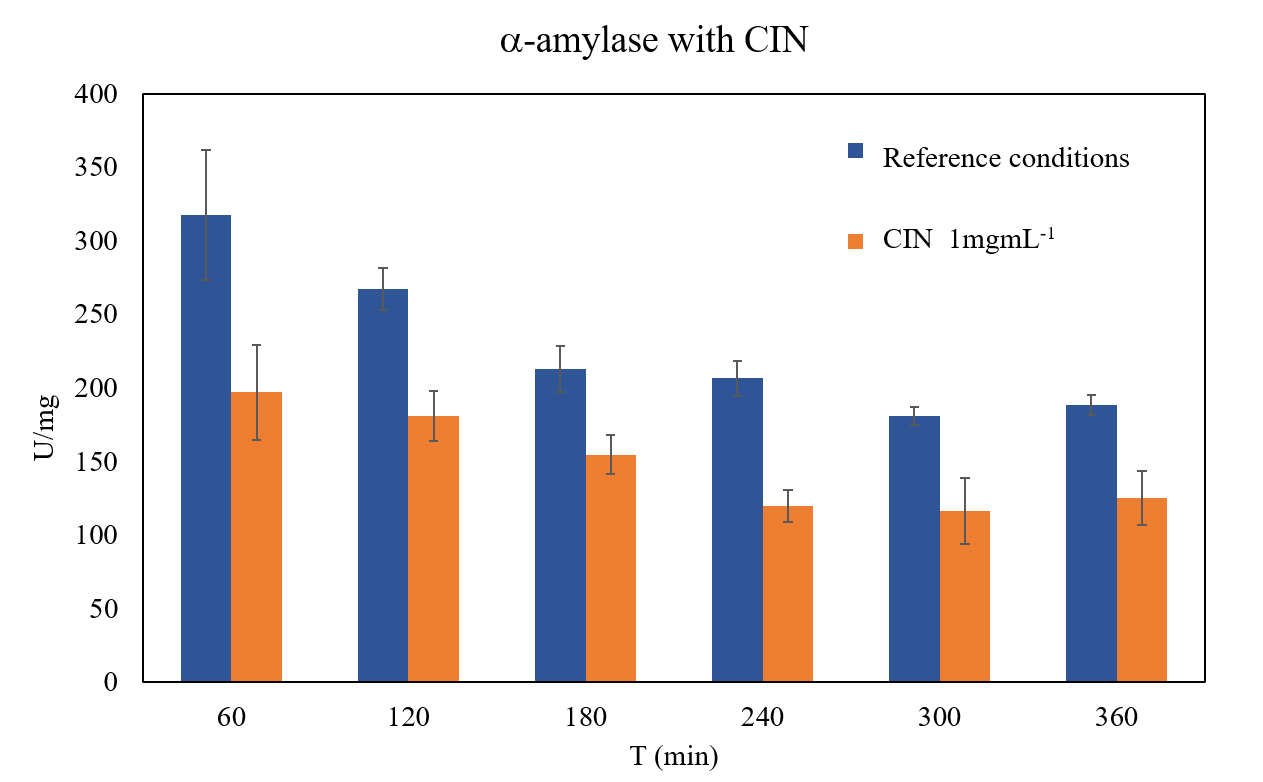

Supplement: Supplementary file 1 [file microorganisms-09-00819-s001.zip › Figure S3.png]
